# Effects of Pre-Cooling on Thermophysiological Responses in Elite Eventing Horses

**DOI:** 10.3390/ani10091664

**Published:** 2020-09-16

**Authors:** Lisa Klous, Esther Siegers, Jan van den Broek, Mireille Folkerts, Nicola Gerrett, Marianne Sloet van Oldruitenborgh-Oosterbaan, Carolien Munsters

**Affiliations:** 1 Department of Human Movement Sciences, Faculty of Behavioural and Movement Sciences, Vrije Universiteit Amsterdam, Amsterdam Movement Sciences, Van der Boechorststraat 7-9, 1081 BT Amsterdam, The Netherlands; l.klous@vu.nl (L.K.); m.a.folkerts@vu.nl (M.F.); n.m.gerrett@vu.nl (N.G.); 2 Department of Clinical Sciences, Faculty of Veterinary Medicine, Utrecht University, Yalelaan 112, 3584 CM Utrecht, The Netherlands; e.w.siegers@uu.nl (E.S.); m.sloet@uu.nl (M.S.v.O.-O.); 3 Department of Population Health Sciences, Utrecht University, Yalelaan 7, 3584 CL Utrecht, The Netherlands; j.vandenBroek@uu.nl; 4 Moxie Sport Analysis & Coaching, Looieind 1, 5469 Erp, The Netherlands

**Keywords:** horses, pre-cooling, rectal temperature, sweat composition, sweat rate

## Abstract

**Simple Summary:**

Horses have a high metabolic capacity for exercise, producing a great deal of heat, and have a small surface area for heat loss. Under limited circumstances, the regulation of heat loss (i.e., across the respiratory tract and by the evaporation of sweat) means heat build-up in the body is reduced. Thermoregulation can be assisted by cooling the horses down to safely perform exercise in thermally challenging environments. The present study showed that pre-cooling (i.e., cooling between the warm-up and exercise performance) slightly reduced the rise in rectal, shoulder and rump skin temperatures of ten international eventing horses during moderately intense canter training in moderate environmental conditions. During the canter training, heart rate, sweat rate and sweat composition were unaffected by pre-cooling. The pre-cooling strategy chosen here was cold-water rinsing for a short period of time (~8 min). Considering the limited time and space at equestrian events, such a pre-cooling strategy could easily be implemented. Reducing heat strain by pre-cooling may potentially improve equine welfare during events.

**Abstract:**

In this study, we examined the effects of pre-cooling on thermophysiological responses in horses exercising in moderate environmental conditions (average wet bulb globe temperature: 18.5 ± 3.8 °C). Ten international eventing horses performed moderate intensity canter training on two separate days, and were either pre-cooled with cold-water rinsing (5–9 °C for 8 ± 3 min; cooling) or were not pre-cooled (control). We determined velocity (V), heart rate (HR), rectal temperature (T_re,_), shoulder and rump skin temperature (T_shoulder_ and T_rump_), plasma lactate concentration (LA), gross sweat loss (GSL), and local sweat rate (LSR), as well as sweat sodium, chloride and potassium concentrations. The effect of pre-cooling on T_re_ was dependent on time; after 20 min of exercise the effect was the largest (estimate: 0.990, 95% likelihood confidence intervals (95% CI): 0.987, 0.993) compared to the control condition, resulting in a lower median T_re_ of 0.3 °C. Skin temperature was also affected by pre-cooling compared to the control condition (T_shoulder_: −3.30 °C, 95% CI: −3.739, −2.867; T_rump_: −2.31 °C, 95% CI: −2.661, −1.967). V, HR, LA, GSL, LSR and sweat composition were not affected by pre-cooling. In conclusion, pre-cooling by cold-water rinsing could increase the margin for heat storage, allowing a longer exercise time before a critical T_re_ is reached and, therefore, could potentially improve equine welfare during competition.

## 1. Introduction

Half the body mass of horses consists of skeletal muscle mass, and consequently, the mass-specific heat load for exercising horses is two- to three-fold higher than for exercising humans, while horses have a low surface-area-to-body mass ratio for heat dissipation (~1:100) compared to humans (~1:40) [1,2,3]. During high-intensity exercise, like eventing, the discrepancy between heat production and dissipation stresses the equine thermoregulatory system [1,2,4,5]. Only under limited circumstances (i.e., low environmental temperature, low exercise intensity, and short duration exercise) does the heat dissipation across the respiratory tract and from the evaporation of sweat from the skin surface prevents thermal strain in horses. However, in most environmental conditions, the potential to develop heat strain during exercise increases significantly because evaporation is impeded [1,4,5]. Therefore, most horses are at risk of exercise-induced hyperthermia when exercising through rapidly increasing core temperature [1,4]. 

To safely perform exercise in challenging environmental conditions, it is beneficial to assist the thermoregulation of the horse by providing cooling [6]. The need for cooling strategies evolved from ensuring the welfare of horses during competitions, commonly held during summer in hot and/or humid environmental conditions. Due to their sensitive gastrointestinal tract, horses are usually cooled externally (i.e., cold-water rinsing) rather than internally (i.e., drinking icy water). In the best case scenario, a cooling strategy lowers core temperature, which is considered as the critical determinant of performance in the heat [7]. However, it is more realistic to expect reductions in skin temperature utilizing external cooling strategies.

Lowering the skin temperature could be beneficial by increasing the temperature gradient between the skin and environment, which facilitates convective heat loss. Several post-cooling studies (i.e., cooling after exercise) have shown an effect of cooling on skin and even core temperatures [6,8,9,10]. Cooling can also be provided prior to exercise (pre-cooling) to increase the margin for heat storage by decreasing pre-exercise core and skin temperatures [11]. In this way, performance may even be influenced, compared to post-cooling. To provide the fastest cooling rate, human research supports the use of cold-water immersion [12,13]. For horses, cold-water immersion is not practical; however, considering the large convective cooling capacity of water, as much surface as possible could be exposed to cold water to still show good effects [6,8].

Eventing is an equine discipline consisting of dressage, cross-country and show jumping on one or three consecutive days. Competitions are held at the national and international level (three-day events), of which the latter ranges from the Concours Complet International level 1 (CCI1*) to the highest level 5 (CCI5*; including the Olympic Games). At the international level, on the first day, a dressage test is performed (a 6-min low intensity technical skills test). On the following day, horses perform a cross-country test, which consists, at the highest level, of a track up to 6270 m including 40 jumping efforts that has to be completed at a velocity (V) of 570 m/min (~9.5 m/s; taking up to 12 min in total). On the third and final day, a show jumping test is performed. Considering the physically challenging cross-country test [2,14,15], pre-cooling may be particularly beneficial there. In a recent practical recommendation for the Olympic Games in Tokyo, The Fédération Equestre Internationale (FEI) already recommends to pre-cool eventing horses before the cross-country test under particular environmental conditions (wet bulb globe temperature (WBGT) >28 °C) [16]. However, to date, no scientific study backs up this recommendation and practical pre-cooling methods for horses have not been studied in-depth.

Equine research has shown that the sudomotor response is the most effective way of losing heat during exercise [5]. The apocrine sweat glands that cover the horse’s entire body surface excrete a sweat solution that is slightly hypertonic to blood [17,18]. Producing large volumes of this hypertonic sweat may therefore have serious implications on overall electrolyte losses. Core temperature is the main driver of sweat production and skin temperature has a modifying effect [19]. The modifying effect of skin temperature is stimulating when skin temperature is high and inhibiting when skin temperature is low. Pre-cooling might lower core and/or skin temperature in horses as it does in humans [20], possibly resulting in lower sweat rates and electrolyte losses. This could lead to the conservation of important electrolytes, alongside an attenuated core and/or skin temperature. 

The main objective of this study was to evaluate the effect of pre-cooling on thermophysiological responses in horses. We hypothesized that pre-cooling by cold-water rinsing would cause a lower heart rate (HR), rectal (T_re_) and skin temperature during exercise. Secondly, we hypothesized that the sweat rate and the excretion of electrolytes in sweat would be reduced during exercise following pre-cooling.

## 2. Materials and Methods

### 2.1. Participants

In setting up the study and related report, the STROBE guidelines were followed whenever possible [21]. The Animal Ethics Committee of Utrecht University approved all procedures (reference number: 5204-1-3, approval date: 19 June 2019). We also obtained the individual horse owner’s informed consent. Ten international Warmblood eventing horses (six geldings, four mares; age: 8.4 ± 2.6 years; body mass: 578 ± 36 kg, height at withers 1.69 ± 0.04 m, and body mass index (BMI): 203.7 ± 8.2 kg/m^2^) participated in this study. Horses were in full training according to their competition level: six horses performed at the CCI1*–2* level (novice horses: age 6.5 ± 0.5 years) and four horses performed at the CCI4*–5* level (advanced horses: age 11.3 ± 1.8 years). All horses were in training for several years (3–10 years) and had at least 5 months of specific conditional training up to their competition level. As the horses were all competing at high international levels in eventing, these horses were assumed to have a high fitness level. During the study (mid-summer in The Netherlands), all horses were in their summer coat (<5 mm). The horses were ridden by their usual rider, housed in individual stables, provided water ad libitum, and fed an individual diet.

### 2.2. Design

The protocol consisted of a moderate intensity canter training on two separate days (with two days in between) in July 2019 in the Netherlands. All canter training sessions were performed on the same 1300-m sand-based track. This type of training was selected to prevent interfering with horses’ individual competition schedules. A cross-over design was used. Horses performed the same moderate intensity canter training on two separate days and were pre-cooled on one occasion (cooling) or not (control). The horses were randomly selected for the cooling or control treatment on the first day and had the other treatment on the second day. Horses were tested at the same time of day (±1 h) in fixed pairs.

The warm-up consisted of a 10-min walk, 10-min trot, 3-min canter (5 m/s) and 3-min walk. Immediately after warming-up, the horses were pre-cooled using 60 L of cold-water (5–9 °C). Cold-water was used because it was previously shown to be an effective post-cooling strategy (water temperature range ~5–9 °C [6,8,9,10]). Water was continuously poured (8 ± 3 min) over the horse’s entire body surface by two researchers with 10 L buckets. As there is limited time and space to pre-cool the horses at equestrian events, this short and practical strategy was chosen.

The canter training started immediately after pre-cooling and consisted of two 4.5-min canter bouts with a 2-min walk in between and ended with a 10-min walking recovery. Novice horses aimed to canter at 6.7 m/s and advanced horses at 7.5 m/s. The exercise intensity was chosen in accordance with their riders and in alliance with their normal conditional training work (performed by experienced elite eventing riders at the CCI5* level) and on the evaluation of previous conditional training sessions (HR and V) of these horses. The chosen velocities were considered as moderate intensity (<V_LA2_ [22]) for these horses.

### 2.3. Measurements

**Figure 1:**
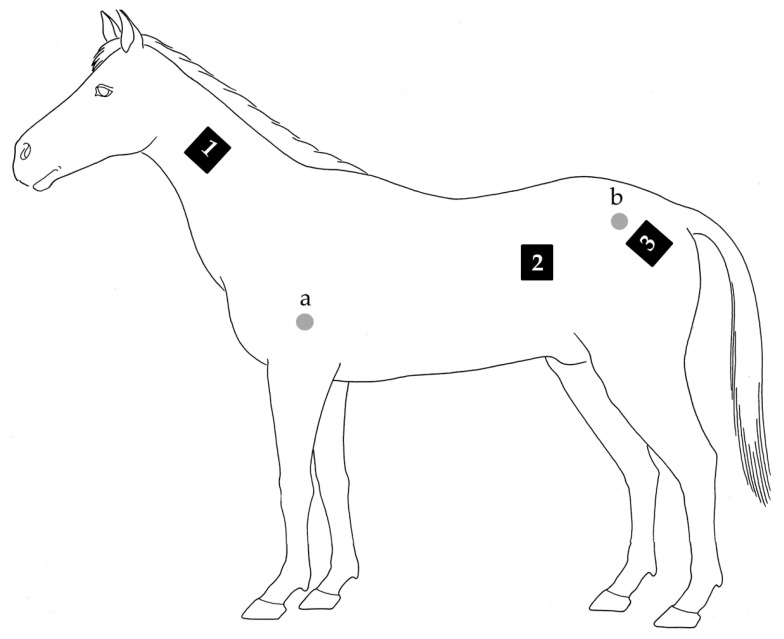
Measurement locations of the skin temperature sensors, represented by the grey cycles (a = shoulder; and b = rump), and absorbent sweat patches, represented by the black rectangles (1 = neck; 2 = barrel; and 3 = rump).

To calculate BMI (i.e., the ratio of height and body mass), the height of the withers was determined for each horse. Body mass of the horses and mass of the tack including the saddle pads and protection boots were measured before saddling and after the canter training (horse scale: custom-made, Breinler International; platform scale: SATEX 34 SA-1 250, Weegtechniek Holland). Feces produced between the first and second weighing moment were collected and weighed. WBGT (QuesTemp 32, 3 M) was obtained before the start of each run. The horses were equipped with a HR monitor with a built-in GPS system at 12 Hz (V800, Polar). A T_re_ sensor (B10014, MSR) was inserted to a depth of 25 cm into the rectum with the datalogger attached to the base of the tail. Skin temperature sensors (i-Button 1922 L, Maxim Integrated) were attached to the shoulder and rump (T_shoulder_ and T_rump_; Figure 1) [23] with removable glue (Bison Kit, Bison International). Gross sweat loss (GSL) was calculated using Equation (1):

GSL = (*m*_horse-post_ − *m*_horse-pre_) + (*m*_tack-post_ − *m*_tack-pre_) − *m*_feces_ (1)

where m represents mass (kg). Local sweat rate (LSR) was assessed by the absorbent patch technique [24]. Three custom-made absorbent patches (25 cm^2^) were attached to the neck with removable glue (Figure 1). LSR was calculated according to Equation (2):

LSR = (*m*_wet_ − *m*_dry_)/t/SA. (2)

Here, $m_{wet}$ refers to mass of the wet patch (g) after the experiment, $m_{dry}$ to the dry mass (g), t represents application time (min) and SA is the surface area (m^2^). Sweat sodium, chloride and potassium were determined by ion-selective electrodes (Cobas 8000, Roche Diagnostics) (Table 1) after centrifuging (1500× *g* for 5 min) the patches in sealed plastic tubes. The patches were analyzed for background concentrations and corrections were made accordingly (sodium −7.9 mmol/L and chloride −6.6 mmol/L). No correction was needed for potassium. Blood samples were taken from the jugular vein within 30 s after the last canter bout and again after 10 min of recovery. Plasma lactate concentration (LA_0_ and LA_10_) was determined with a portable hand-held lactate measurement device (Lactate Pro2, Arkray Inc.) [25].

### 2.4. Statistical Methods

Continuous recorded data were transformed to 1-min averages. To check whether the normality assumption was reasonable, we made normality probability plots of the residuals. If the normality assumption did not hold, the data was log-transformed to obtain a normal distribution (transformed T_re_, LA_0_, and LA_10_). If data were transformed, the statistical outcome medians rather than the means were reported [26]. The data of the training session following pre-cooling or control were analyzed. HR, T_re_, T_shoulder_, and T_rump_ were analyzed using a mixed effect model with horse as a random effect and with cooling, sex, age, BMI, WBGT, day, time, and time *x* cooling interactions as fixed effects. This was called the starting model. Due to the strong relationship between the competition level and age, only age was included in the starting model. Model reduction was done using Akaike’s information criterion (AIC) using R (R: A Language and Environment for Statistical Computing, 2019). The model for which there was no further reduction possible was taken as the final model, and the remaining factors were considered important [27]. The 95% profile likelihood confidence intervals (95% CI) were reported where there was an important effect.

Mixed effect models were used to analyze V, LA_0_, LA_10_, GSL, and LSR, as well as sweat sodium, chloride, and potassium concentrations with random horse effects and sex, age, BMI, WBGT, day, and pre-cooling as fixed effects (i.e., the starting model). Model reduction was again done using AIC in IBM SPSS Statistics 26.0.

## 3. Results

The control condition HR data of one horse were missing due to technical issues. In addition, T_shoulder_ data of two horses (cooling: 1; and control: 1) was missing because the sensor fell off during the training. As data collection took place on two separate days, differences in WBGT occurred (day 1: 15.9 ± 0.4 °C; day 2: 21.1± 3.9 °C). The velocities did not differ between cooling and control conditions (Table 2).

### 3.1. Heart Rate

**Figure 2:**
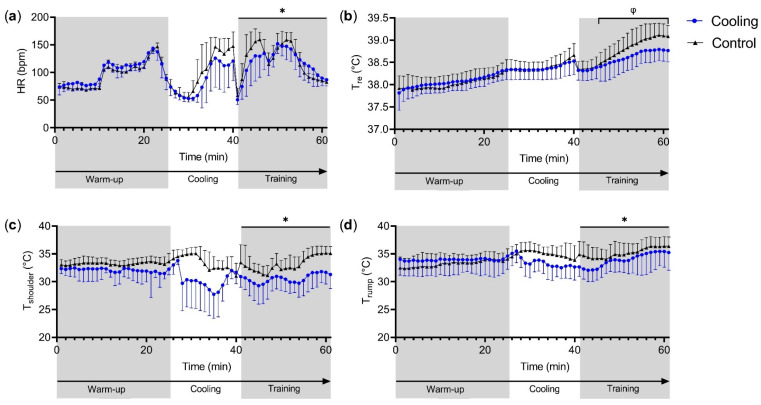
Mean and standard deviation of the physiological responses of ten international eventing horses in the Netherlands (average wet bulb globe temperature: 18.5 ± 3.8 °C). The data are shown for the warm-up and canter training with either pre-cooling (cooling) or not (control) in between. (**a**) Heart rate (HR); (**b**) rectal temperature (T_re_); (**c**) shoulder skin temperature (T_shoulder_); and (**d**) rump skin temperature (T_rump_). * Denotes an effect of cooling. ^φ^ Denotes an interaction effect of time x cooling.

Age, day, and time had an effect on HR during training. Older horses showed on average a higher HR (3 bpm, 95% CI: 2.197, 4.062). HR was lower on the second compared to the first day (estimate: −6 bpm, 95% CI: −10.333, −2.555) and there were time effects. Pre-cooling showed a tendency to an average HR decrease (−4 bpm, 95% CI: −7.738, 0.039) (Figure 2, Supplementary Table S1).

### 3.2. Rectal Temperature

Every degree increase in WBGT increased median T_re_ by 1.00 °C (95% CI: 1.0008, 1.0012). At the second and hotter testing day, the median T_re_ was 0.99 °C higher (95% CI: 0.994, 0.997). There were time effects and interactions of time x pre-cooling effects. From 6 min on in training, pre-cooling attenuated the increase in T_re_ compared to the control condition, with the largest effect after 20 min: T_re_ was 38.9 ± 0.3 °C in horses that were pre-cooled and 39.1 ± 0.2 °C in the control condition (0.990, 95% CI 0.987, 0.993) (Figure 2, Supplementary Table S2).

### 3.3. Shoulder Skin Temperature

Sex, age, WBGT, time, and pre-cooling affected the T_shoulder_ results. Geldings showed, on average, a lower T_shoulder_ (−0.96 °C, 95% CI: −1.459, −0.459), older horses showed a higher average T_shoulder_ (0.15 °C, 95% CI: 0.058, 0.233) and higher WBGT values led to higher T_shoulder_ values (0.14 °C, 95% CI: 0.082, 0.199). Following pre-cooling, the horses showed, on average, a lower T_shoulder_ (−3.30 °C, 95% CI: −3.739, −2.867), and there were time effects (Figure 2, Supplementary Table S3).

### 3.4. Rump Skin Temperature

WBGT, day, time, and pre-cooling affected T_rump_. Higher WBGT values led to, on average, higher T_rump_ values (0.55 °C, 95% CI: 0.463, 0.645). T_rump_ was lower on the second testing day (−1.39 °C, 95% CI: −1.958, −0.822). Pre-cooling caused, on average, a lower T_rump_ (−2.31 °C, 95% CI: −2.661, −1.967), and there were time effects (Figure 2, Supplementary Table S4).

### 3.5. Plasma Lactate

There was no effect of pre-cooling on LA_0_ (cooling: 1.7 ± 0.6 mmol/L; control: 1.6 ± 0.3 mmol/L) and LA_10_ (cooling: 0.8 ± 0.1 mmol/L; control: 0.9 ± 0.1 mmol/L).

### 3.6. Sweating Response

None of the factors affected GSL (cooling: 5.18 ± 1.55 L; control: 5.53 ± 1.73 L) and LSR on all three measurement locations and their corresponding sodium, chloride and potassium concentrations (Table 3).

## 4. Discussion

To our knowledge, this is the first study to assess the thermophysiological effects of pre-cooling on horses. Despite the moderate intensity exercise and moderate environmental conditions elicited, we observed rises in thermophysiological variables (Figure 2, Table 3). Most importantly, pre-cooling by cold-water rinsing directly after warming-up led to a smaller rise in T_re_, T_shoulder_, and T_rump_ during training compared to the control condition. These attenuated rises can be used to increase the margin for heat storage, allowing a longer exercise time before a critical T_re_ (for horses most likely T_re_ ~41 °C [1,4,28,29]) is reached. This may help to optimize equine welfare during eventing competitions and may potentially be beneficial for performance on the cross-country test, even in moderate environmental conditions. In addition, the environmental conditions and individual horse characteristics (like sex, age, and breed) should be taken into account as these affected T_re_, T_shoulder_, and/or T_rum__p_.

### 4.1. Testing Day

Minimal effects of testing day were expected because a cross-over design was used. However, for the second and warmer testing day (WBGT day 1: 15.9 ± 0.4 °C; day 2: 21.1 ± 3.9 °C), we found significantly lower HR and T_rump_ values but higher T_re_ values (Supplementary Tables S1–S4). Based on visual observations, the lower HR appeared to be caused by the fact that the horses were more habituated to the situation the second time, which caused less arousal. The higher environmental temperature at the second testing day likely impaired the heat dissipation by the smaller temperature difference between the skin and environment, causing larger heat storage and consequently a higher overall T_re_. It is plausible to assume that, on the cooler day, more heat was lost via skin conduction, heating up the skin.

In the present study, there was a (non-significant) difference between the testing days in GSL; 5.89 ± 1.66 L at the second and warmer testing day and 4.83 ± 1.83 L on day 1 (*n* = 10). This likely supported cooling the skin down and attenuating the rise in T_rump_. Such differences in physiological responses between testing days are not surprising considering previous equine research, concluding that the number of exertional heat illness cases was significantly higher when WBGT was 20–23.9 °C (~day 2 of the present study) compared to <20 °C (~day 1 of the present study) [30]. 

In addition, physiological responses were compared in different environmental conditions [4,17,28,31]. McCutcheon et al. [17] concluded that LSR and sweat electrolyte concentrations were largely reflected by a higher environmental temperature (20 °C to 32–34 °C). Likewise, Geor et al. [4] observed significantly higher rates of heat storage in hot-dry (WBGT: 24.6 ± 0.3 °C) and hot-humid conditions (WBGT: 24.7 ± 0.3 °C) compared to cold-dry conditions (WBGT: 16.6 ± 0.2 °C). Therefore, the ~5 °C difference in WBGT shown here, even in cool to moderate environmental conditions, can have a large effect on rates of heat storage in eventing horses at competition days or during moderate to intense training sessions.

Despite balancing the order of testing, such temperature-dependent observations should be taken into consideration when interpreting our results, which were collected on two days that differed regarding the environmental conditions. Interestingly, in previous research the required evaporation (E_req_) already exceeded the maximal evaporative heat loss (E_max_) in cool-dry conditions (WBGT: 16.6 ± 0.2 °C) [4]. By definition, this represents un-compensable heat stress. Assuming that the un-compensable heat stress also applies to the present study on both testing days, it is likely that the conditions were at least similar regarding compensability (i.e., the effectiveness of thermoregulation). From a practical point of view, this should be highlighted to eventing riders and trainers, as it is commonly believed that these conditions are not challenging for eventing horses.

### 4.2. Thermophysiological Responses

In the present study, the rise in T_re_ during training was smaller following pre-cooling than without a pre-cooling strategy (control; Figure 2). As pre-cooling is intended to increase the margin for the rise in core temperature [20], our pre-cooling strategy appeared to be effective [1,32,33]. As expected, the largest absolute effect of pre-cooling was observed for both T_shoulder_ and T_rump_ (~2–3 °C; Figure 2), which is due to the cold-water rinsing of the skin. A larger difference between core and skin temperature allows for more heat dissipation by conduction, which may reduce heat strain [1]. The largest effect of the pre-cooling strategy on T_re_, an indicator of core temperature, which is considered the critical determinant for performance in the heat [7], was only 1% or 0.3 °C here (Supplementary Table S2). Although, the normal T_re_ range of a horse is narrow (variation ~1 °C [34]), from a physiological point of view, such a reduction in T_re_ may seem small. In particular, when considering that alongside pre-cooling, multiple factors, like environmental conditions, affected T_re_ to the same extent.

However, only a few studies evaluated the horses under field conditions in a comparable situation where they actually train and compete. In relation to cooling methods, this may be important as the mass of the rider and saddle, and loss of cooling surface area due to the saddle can affect the thermophysiological responses of a horse [31]. In a field study by Hargreaves et al. [31], submaximal exercise in horses was evaluated in hot-humid (HH; environmental temperature 31.3 ± 0.9 °C) and cool-dry (CD; 17.6 ± 0.4 °C) circumstances. While in controlled laboratory settings, the environmental conditions largely affected the physiological parameters as discussed above, Hargreaves et al. [31] observed that, directly after cantering, T_re_ was 39.0 °C in HH and 38.5 °C in CD conditions. In light of this relatively small T_re_ difference (~0.5 °C) between conditions, lowering T_re_ by 0.3 °C as in the present study may be physiologically relevant.

In another study by Marlin et al. [6] following high-intensity exercise in hot-humid environments, end-exercise T_re_ was 39.3 ± 0.3 °C, while simultaneously pulmonary artery temperature (T_pa_) was 42.3 ± 0.4 °C. After the first 30-s post-cooling period (water temperature ~6 °C), T_pa_ decreased directly while T_re_ continued to increase (to 40.1 ± 0.2 °C). After 2 min of cooling, T_re_ showed a gradual decrease during the rest of the total cooling period of 6 min (end T_re_ 39.3 ± 0.3 °C, while end T_pa_ was 38.2 ± 0.2 °C). The decreases correspond to 12 °C/h (0.2 °C/min) for T_re_, while T_pa_ decreased with 0.8 ± 0.1 °C/min.

In a more recent study, five cooling methods in a hot-humid environment (WBGT temperature: 31.8 ± 0.1 °C) were evaluated. These horses showed the largest decrease in T_pa_ and T_re_ using a shower method (tap water of ~26 °C for 30 min), compared to the intermittent application of cold-water (16 L of ~10 °C water every 3 min for a total of 30 min) [8]. When the horses were showered after the intense training session, T_re_ decreased from 39.7 ± 0.6 °C to 38.6 ± 0.4 °C in 30 min. This is a decrease in T_re_ of 0.04 °C/min, corresponding to a total decrease of 0.29 °C after 8 min of cooling (which is the average pre-cooling time of the present study). Utilizing the showering method, T_pa_ decreased just below 39.0 °C within 2.1 ± 0.6 min, and, after 9 min, T_pa_ decreased to baseline temperatures (T_pa_ ~38 °C). This would reflect a T_pa_ decrease of 0.44 °C/min.

In the study of Kohn et al. [10], where the horses were washed with cold-water post-exercise a 0.05 °C/min decrease in T_re_ was seen in the first 15 min. At the same time, T_pa_ decreased with a rate of 0.25 °C/min. The lag in reduction of T_re_ compared to T_pa_ represents a delay in distribution of heat to peripheral body tissues or may reflect the heat of the hind limbs transferred to the rectum [1,35]. It is important for horse riders, trainers, and owners to understand that T_re_ is not as indicative of the actual core temperature (i.e., temperature of the internal organs) as is sometimes assumed, due to the lag in T_re_. An increase in T_re_ commonly corresponds to a larger increase in T_pa_ and muscle temperature [4,6,8,10], and this should be taken into account when predicting the core temperature and assessing heat strain in horses.

As shown in the studies mentioned above, post-cooling methods facilitate more heat dissipation compared to the conditions without cooling. Effective post-cooling methods appear to result in a T_re_ decrease of between 0.04–0.2 °C/min [6,8,10]. The present study was performed under field conditions and the horses were cooled prior to exercise instead of post-exercise, resulting in an increase in T_re_ as heat is still produced due to exercise. The cooling durations elicited in the abovementioned studies largely exceed the time available for pre-cooling horses at equestrians events. In addition, saddled horses have a restricted cooling surface area, compared to non-ridden horses.

Here, we observed an attenuated increase of 0.3 °C following ~8 min of pre-cooling (~0.04 °C/min) in saddled horses compared to the control condition. This seemingly small difference in T_re_ can still be biological effective, especially as it shows a similar cooling rate in T_re_ compared to other post-cooling studies [6,8,10]. It would have been interesting to measure T_pa_ in these horses as larger temperature effects are typically visible in T_pa_ compared to T_re_. Unfortunately this was not possible in these horses under field conditions. As there is evidence of a dose-response relationship between pre-cooling volume and ensuing physiological outcomes [36], future research should investigate a more aggressive pre-cooling type (i.e., larger volume or longer duration). Nevertheless, small reductions in heat strain could already help improve horse welfare during equestrian events.

On the other hand, as the potential thermophysiological benefit of pre-cooling may be relatively small, the advantages and disadvantages of pre-cooling should be considered carefully. The potential detrimental effects of pre-cooling could arise from counteracting the increase in muscle temperature, elicited by warming-up. Elevated muscle temperatures allow for increases in the muscle metabolism, that positively affect performance. However, a T_re_ of 41 °C is the upper limit, at that moment muscle temperature is already much higher, and it is likely that muscle denaturation will occur [37,38]. This should absolutely be avoided at all times.

Pre-cooling may not be beneficial prior to short-duration exercise, which heavily relies on muscle metabolism. However, pre-cooling may be beneficial for longer-duration exercise (>5 min), with a higher thermoregulatory burden. We observed a significant effect of pre-cooling on T_re_ from 6 min into exercise (Supplementary Table S2) and the largest effect after 20 min of exercise, supporting this theory.

Anecdotal evidence indicates that in practice riders and trainers already pre-cool their eventing horses before the cross-country test, while others believe that pre-cooling causes myopathies and muscle cramps. Previous post-cooling studies have not observed muscle stiffness or discomfort of the horses [6,9]. Neither were any adverse effects of pre-cooling found during nor following this study. As core temperature is the main driver of sweating [19,39], the non-significant different GSL is most likely explained by the small differences in T_re_ during training following or without cooling (Figure 2). Locally, pre-cooling did not affect the sweat production or sweat composition (Table 3). Since LSR affects sweat composition [17] and was not different between conditions, this likely explains the absence of differences in sweat composition. Future research is still required to fully understand the thermophysiological impact of pre-cooling on horses.

### 4.3. Exceptional Individual Responses

Above, we mentioned that alongside pre-cooling, multiple other factors affected the parameters that we examined here. The individual horse is one of these factors. Following pre-cooling, one horse unexpectedly showed a higher T_re_ compared to the control condition (Figure S1), whereas the other nine horses showed a response similar to the group average. Even though day-to-day variation in T_re_ is a well-known phenomenon [17], the rise in T_re_ during training was also comparable (cooling: +0.50 °C, control: +0.51 °C). For this horse, the external (V) and internal (LA_0_ and LA_10_) workload indicators were similar between conditions. The pre-cooling protocol may not have affected thermoregulation in this horse (i.e., a poor responder). Considering the doseresponse relation of pre-cooling volume and ensuing physiological outcomes [36], it would have been interesting to investigate a more aggressive pre-cooling type (i.e., a larger volume of water). Secondly, one horse (a gelding, 13 years, and CCI4–5* level) had a final T_re_ of 39.6 °C (+1.3 °C rise) in the control condition, which is unexpectedly high considering the environmental conditions. The pre-cooling strategy was effective in reducing the end-exercise T_re_ for this horse by −0.5 °C. If this horse participates in events in more challenging conditions, T_re_ will potentially rise up to dangerous values, emphasizing the importance of collecting individual data regarding thermoregulatory profiling, as is recommended in humans [40].

### 4.4. Limitations

A significant limitation of the present study is that the environmental conditions on both testing days differed considerably, which is discussed in detail above. The researchers attempted to minimize the differences in environmental conditions by choosing stable summer weather. However, this will always be challenging in field studies. Another potential limitation may be that the findings only apply to moderate environmental conditions and exercise intensity used in the present study. The effect of pre-cooling on the measured variables may have been larger if a high intensity exercise protocol in hot and/or humid conditions was used [20]. However, we used privately-owned high-performance horses, and thus the circumstances had to be within acceptable limits. In addition, the included horses here all compete in the highest international eventing levels, and thus, were assumed to have high fitness levels. Previous research showed larger performance benefits of pre-cooling in humans with a high compared to a low aerobic fitness level [20]. Consequently, the benefit of pre-cooling could have been less in relatively unfit horses. Therefore, evaluating the effects of pre-cooling in an controlled and more challenging environment, at different exercise intensities and/or with horses of different levels of fitness, would be interesting for further research.

Considering the data collection, it would have been interesting to measure a closer indicator of the actual core temperature (i.e., the temperature of the internal organs) than T_re_. However, measuring the pulmonary artery (T_pa_) or muscle temperature was not feasible in this field study. Additionally, at equestrian events, T_re_ is commonly measured, allowing for an easy translation to practice. To further improve our understanding of the impact of pre-cooling on horses, future research should include a performance measure (i.e., the time to complete a certain distance) alongside a thermoregulatory observational part.

## 5. Conclusions

Pre-cooling horses by whole-body cold-water rinsing, directly before moderate intensity training, led to a slightly smaller rise in rectal and skin temperatures in moderate environmental conditions. Considering the relatively small, and late (after 6 min), impact on the thermophysiological parameters, pre-cooling may be most beneficial in moderate- to longer-duration exercise with a high thermoregulatory burden, such as the cross-country test in the eventing discipline. Understanding the individual responses to pre-cooling is important for optimising welfare, and possibly improving performance during training and competition in challenging environmental conditions.

## Figures and Tables

**Table 1:** Accuracy of the analyzers for the electrolyte concentrations in sweat.

Component	LOD (mmol/L)	CV (%)	Sample Volume (μL)
Sodium	0.4	<1.0	15
Chloride	0.4	<2.2	15
Potassium	0.2	1.0–1.3	15

LOD: Limit of detection; CV: coefficient of variation.

**Table 2:** Mean and standard deviation of the performed velocities of ten international eventing horses in the Netherlands during the warm-up and training following pre-cooling (cooling) or not (control) (average wet bulb globe temperature: 18.5 ± 3.8 °C). The stages are chronologically ordered from top to bottom.

Stage	Gait	V (m/s)	
		Cooling	Control
Warm-up	Walk	1.6 ± 0.2	1.6 ± 0.1
	Trot	3.3 ± 0.3	3.3 ± 0.3
	Canter	4.6 ± 1.2	4.8 ± 1.4
	Walk	0.7 ± 0.3	0.7 ± 0.4
Pre-cooling		Yes	No
Canter training	Canter I	7.0 ± 0.1	7.1 ± 0.2
	Canter II	6.8 ± 2.2	6.9 ± 0.7
	Recovery	1.6 ± 0.0	1.6 ± 0.1

V: velocity; Canter I: first 4.5-min canter bout; Canter II: second 4.5-min canter bout.

**Table 3:** Mean and standard deviation of the local sweating response and electrolyte concentrations at three skin sites of ten international eventing horses in the Netherlands during the warm-up and canter training following pre-cooling (cooling) or not (control) (average wet bulb globe temperature: 18.5 ± 3.8 °C).

Stage	Skin Site	Cooling				Control			
		LSR (mL/m^2^/min)	Sodium (mmol/L)	Chloride (mmol/L)	Potassium (mmol/L)	LSR (mL/m^2^/min)	Sodium (mmol/L)	Chloride (mmol/L)	Potassium (mmol/L)
Warming-up	Neck	7.6 ± 3.3	92.0 ± 16.8	152.9 ± 28.8	65.0 ± 13.9	6.8 ± 4.7	87.1 ± 34.3	148.0 ± 43.6	67.7 ± 18.3
	Barrel	5.6 ± 2.6	105.1 ± 23.9	181.6 ± 41.9	57.7 ± 17.5	5.2 ± 3.6	91.4 ± 21.4	150.3 ± 41.5	68.6 ± 12.0
	Rump	5.0 ± 4.8	109.0 ± 23.4	187.6 ± 32.5	76.3 ± 7.4	3.7 ± 2.1	77.6 ± 40.8	153.4 ± 94.0	67.3 ± 43.0
Canter training	Neck	13.0 ± 3.6	99.1 ± 20.8	137.8 ± 46.1	48.5 ± 18.7	14.1 ± 4.7	95.1 ± 26.9	132.2 ± 43.6	45.0 ± 9.0
	Barrel	10.4 ± 3.1	89.0 ± 16.7	124.1 ± 33.8	43.3 ± 15.8	12.9 ± 2.7	102.8 ± 17.9	145.9 ± 26.8	51.1 ± 9.5
	Rump	16.8 ± 7.1	77.6 ± 39.3	103.6 ± 70.5	35.9 ± 29.0	15.3 ± 5.0	102.8 ± 29.8	169.5 ± 46.1	66.1 ± 16.1

LSR: Local sweat rate.

## Supplementary Materials

The following are available online at https://www.mdpi.com/2076-2615/10/9/1664/s1. Table S1: Results of the final mixed model regarding heart rate. Table S2: Results of the final mixed model regarding rectal temperature. Table S3: Results of the final mixed model regarding shoulder skin temperature. Table S4: Results of the final mixed model regarding rump skin temperature. Figure S1: Deep rectal temperature response.
