# Supplementary material for: Effects of Pre-Cooling on Thermophysiological Responses in Elite Eventing Horses
Source: Animals (Basel). 2020 Sep 16;10(9):1664. doi: 10.3390/ani10091664 (PMC7552184; doi:10.3390/ani10091664)
Supplement: Supplementary file 1 [file animals-10-01664-s001.zip › Supplementary Figure S1.docx]

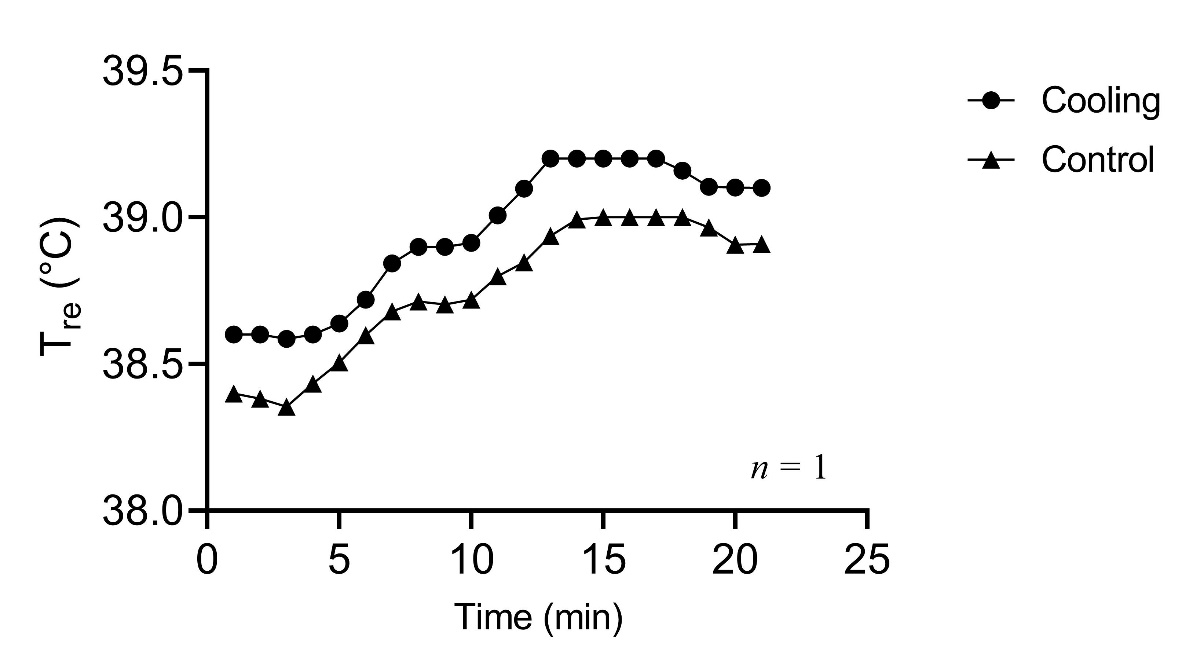


**Figure S1.** The exceptional deep rectal temperature (T_re_) response of one international eventing horse (i.e. opposite T_re_ response in both conditions from the other 9 horses) during the canter training in The Netherlands (wet bulb globe temperature: 18.5 ± 3.8 °C) in the pre-cooling and control condition.
