# Supplementary material for: Effects of Pre-Cooling on Thermophysiological Responses in Elite Eventing Horses
Source: Animals (Basel). 2020 Sep 16;10(9):1664. doi: 10.3390/ani10091664 (PMC7552184; doi:10.3390/ani10091664)
Supplement: Supplementary file 1 [file animals-10-01664-s001.zip › Supplementary Table S4.docx]

**Supplementary Table S4.** Results of the final mixed effect model regarding rump skin temperature (T_rump_) in relation to sex, age, body mass index (BMI), wet bulb globe temperature (WBGT), day, time, cooling and their interactions of ten international eventing horses in the Netherlands. As reference values mares, first day, first min (time) and control condition (no cooling) were taken.

| **T_rump_** | **Estimates of effect** | **95% CI** |
| --- | --- | --- |
| Fixed effects |  |  |
| Intercept | 25.581 | 23.862, 27.230 |
| Sex |  |  |
| Age |  |  |
| BMI |  |  |
| WBGT | 0.554 | 0.463, 0.645 |
| Day | -1.390 | -1.958, -0.822 |
| Time |  |  |
| Min 1 | - | - |
| Min 2 | -0.568 | -1.585, 0.449 |
| Min 3 | -1.044 | -2.061, -0.027 |
| Min 4 | -1.314 | -2.331 -0.297 |
| Min 5 | -1.303 | -2.320, -0.286 |
| Min 6 | -1.005 | -2.022, 0.012 |
| Min 7 | -0.621 | -1.637, 0.396 |
| Min 8 | 0.046 | -0.971, 1.062 |
| Min 9 | 0.387 | -0.631, 1.404 |
| Min 10 | 0.216 | -0.801, 1.233 |
| Min 11 | 0.118 | -0.899, 1.135 |
| Min 12 | 0.239 | -0.778, 1.256 |
| Min 13 | 0.487 | -0.530, 1.504 |
| Min 14 | 1.009 | -0.008, 2.026 |
| Min 15 | 1.614 | 0.597, 2.631 |
| Min 16 | 1.939 | 0.922, 2.956 |
| Min 17 | 2.139 | 1.122, 3.156 |
| Min 18 | 2.110 | 1.093, 3.127 |
| Min 19 | 2.051 | 1.043, 3.077 |
| Min 20 | 2.066 | 1.049, 3.083 |
| Min 21 | 1.976 | 0.959, 2.993 |
| Cooling | -2.314 | -2.662, -1.967 |
| Time x cooling |  |  |
| Random effects |  |  |
| Horse | 1.146 | 0.718, 1.829 |

CI: Confidence interval.
